# Sodium Content of Street and Restaurant Foods in Nigeria: A Multilevel Analysis of Food System Determinants

**DOI:** 10.3390/nu18142348

**Published:** 2026-07-17

**Authors:** Adedayo E. Ojo, Katrina R. Kissock, Gabriel L. Shedul, Ikechukwu A. Orji, Guhan Iyer, Vanessa O. Alfa, Valerie M. Graham, Clementina E. Okoro, Mercy Ikechukwu-Orji, Henry U. EkeChi, Chisom Obiezu-Umeh, Erica L. Jamro, Sanne A. E. Peters, Diederick E. Grobbee, Kylie Howes, Alexandra Jones, Fraser Taylor, Linda Van Horn, Lisa R. Hirschhorn, Bruce Neal, Dike B. Ojji, Mark D. Huffman

**Affiliations:** 1 Cardiovascular Research Centre, University of Abuja and University of Abuja Teaching Hospital, Gwagwalada, Abuja 902101, Nigeria; 2 Julius Center for Health Sciences and Primary Care, University Medical Center Utrecht, Utrecht University, 3584 CG Utrecht, The Netherlands; 3 The George Institute for Global Health, University of New South Wales, Sydney, NSW 2031, Australia; 4 Department of Family Medicine, University of Abuja Teaching Hospital, Gwagwalada, Abuja 902101, Nigeria; 5 Department of Medical Social Sciences, Feinberg School of Medicine, Northwestern University, Chicago, IL 60611, USA; 6 Department of Medicine, Washington University in St. Louis, St. Louis, MO 63110, USA; 7 Department of Nutrition, Federal Ministry of Budget and Economic Planning, Abuja 900211, Nigeria; 8 School of Public Health, Imperial College London, London SW7 2BX, UK; 9 Department of Preventive Medicine, Northwestern University, Chicago, IL 60611, USA; 10 Havey Institute for Global Health, Feinberg School of Medicine, Northwestern University, Chicago, IL 60611, USA; 11 Department of Medicine, Faculty of Clinical Sciences, University of Abuja, Gwagwalada, Abuja 900211, Nigeria; 12 Bursky School of Public Health, Washington University in St. Louis, St. Louis, MO 63130, USA

**Keywords:** street foods, restaurant foods, dietary sodium, sodium reduction, food environment, Nigeria

## Abstract

**Background/Objectives**: Street and restaurant foods constitute a major component of daily diets in low-resource settings due to their affordability, accessibility, and convenience. However, they are frequently characterised by high sodium content, posing significant public health risks, particularly for hypertension and cardiovascular diseases. Empirical data on sodium levels in Nigerian street and restaurant foods remain limited. This study assessed the sodium content of commonly consumed street and restaurant foods in Kano and Ogun States and the Federal Capital Territory in Nigeria using a multilevel analytical framework. **Methods**: A total of 2583 food samples were collected from street vendors and three categories of restaurants (self-service, quick-service, and hotel restaurants) between August and October 2024. Sodium concentration was measured using a validated digital salt meter. In all three states, rice-based dishes were the most commonly available street and restaurant foods. **Results**: More than half (55.4%) were classified as high-sodium (>600 mg/100 g), while only 4.1% were classified as low-sodium (<120 mg/100 g). Median sodium content was highest in the FCT (720 mg/100 g), followed by Kano (650 mg/100 g) and Ogun (600 mg/100 g). Soups and stews had the highest median sodium levels (1360 mg/100 g), with several traditional dishes exceeding 1000 mg/100 g. Multilevel mixed-effects modelling demonstrated that sodium exposure was driven primarily by food type (*p* < 0.001) and vendor environment (*p* = 0.009), rather than geographic location (state effect: *p* = 0.217), indicating structurally embedded issues with sodium content throughout the Nigerian food system. **Conclusions**: These findings support multilevel sodium reduction interventions targeting high-sodium food categories, vendor preparation practices, and strengthened surveillance to monitor progress toward World Health Organization population sodium reduction targets.

## 1. Introduction

Excessive dietary sodium intake is a major public health concern and a well-established, modifiable risk factor for non-communicable diseases (NCDs), particularly hypertension and cardiovascular diseases (CVDs) [1,2,3]. Globally, NCDs account for a substantial proportion of premature mortality, with the burden disproportionately affecting low- and middle-income countries (LMICs) [4]. Projections indicate that, without effective prevention strategies, deaths attributable to NCDs will continue to rise over the coming decades [5]. In Sub-Saharan Africa, NCDs are responsible for approximately 37% of all deaths, reflecting a large and rapidly growing burden driven by urbanisation, dietary transitions, and limited healthcare capacity for chronic disease prevention and management [6].

In Nigeria, the increasing prevalence of NCDs like hypertension and CVDs is closely linked to unhealthy dietary patterns [7]. Evidence from national estimates and empirical studies indicates that sodium intake in Nigeria consistently exceeds recommended levels, largely due to dietary practices involving highly salted foods, processed snacks, and sodium-rich seasonings commonly used in traditional cooking [8,9]. The findings from the Nigeria Sodium Study (NaSS), a nationally representative population-based survey, further highlight the magnitude of this public health concern with a reported average daily sodium intake of 3876 mg, nearly double the World Health Organisation’s (WHO) recommended maximum of 2000 mg/day of sodium (equivalent to 5 g of salt) [9]. This level of sodium intake underscores the need for population-level strategies to reduce dietary sodium and address the growing burden of hypertension and cardiovascular disease in Nigeria. In addition, findings from the Nigeria Sodium Study indicate that foods obtained from street vendors and restaurants contribute substantially to dietary sodium intake among Nigerian adults, accounting for approximately 20% of total sodium consumption [9]. Given the widespread reliance on prepared foods outside the home, these food environments represent important targets for sodium reduction interventions and public health policy [10,11].

Reducing population-level sodium intake is widely recognised as a public health priority both globally and in Nigeria [12,13,14]. To support countries in implementing comprehensive sodium-reduction strategies, the WHO developed the SHAKE technical package: Surveillance, Harness industry, Adopt standards for labelling and marketing, Knowledge, and Environment [15]. Central to this framework is the surveillance pillar, which emphasises the generation of robust, context-specific data on dietary sodium sources to guide policy and intervention design. In Nigeria, the Federal Ministry of Health and Social Welfare (FMoHSW) introduced the National Multi-Sectoral Action Plan (NMSAP) in 2019 to address NCD prevention and control through public awareness campaigns, regulatory actions, and capacity-building initiatives [16]. These efforts have been further strengthened by the National Policy on Food Safety and Quality and its Implementation Plan, as well as the recently developed National Guidelines for Sodium Reduction, which provide a framework for reducing sodium levels in the food supply and supporting population-level dietary sodium reduction [17,18]. Collectively, these initiatives align with the WHO Global Action Plan target of a 30% reduction in population sodium intake from 2015 to 2030 [19].

Street and restaurant foods play a central role in Nigerian diets due to their affordability, accessibility, convenience, and wide variety [10,20,21]. Informal food environments such as street food settings are often underrepresented in national dietary surveillance systems, limiting the availability of evidence needed to inform targeted regulatory and programmatic actions [11]. For the purposes of this study, street foods are defined as ready-to-eat foods prepared and sold by vendors in public spaces, such as markets, roadsides, and motor parks, without a fixed indoor food preparation facility. Restaurant foods are defined as meals prepared and served within fixed-premises establishments, including self-service restaurants, quick-service restaurants, and hotel restaurants.

Nigeria has a population of approximately 240 million, with an annual growth rate of about 2.5% [22]. More than three-quarters of the population reside in towns and cities [23], and street and restaurant foods constitute a significant component of the national food system, with vendors widely distributed across both urban and rural settings. Despite their widespread consumption, there is currently no specific legislation regulating the sodium content of street and restaurants foods in Nigeria. These foods are commonly sold in open markets, roadside stalls, and by mobile vendors [24].

Addressing this evidence gap is essential to strengthening sodium surveillance and supporting the implementation of WHO-recommended sodium-reduction strategies, particularly under the surveillance component of these frameworks [15]. Beyond Nigeria, the findings of this study have direct implications for sub-Saharan Africa and other low- and middle-income regions where street and restaurant foods constitute a major component of daily dietary intake, sodium reduction surveillance systems remain underdeveloped, and the burden of diet-related non-communicable diseases is rapidly increasing. The evidence generated here provides a model for regional sodium assessment approaches applicable across diverse informal food environments. Therefore, this study aimed to assess the sodium content of commonly consumed street and restaurant foods in three states of Nigeria, the Federal Capital Territory, Kano State, and Ogun State, to generate context-specific evidence to inform policy development and public health interventions aimed at reducing dietary sodium intake and improving population health.

## 2. Materials and Methods

This study was conducted in four local government areas (LGAs) per state across three states: the Federal Capital Territory (Federal Capital Territory Municipal Area Council, Bwari, Kuje, and Gwagwalada), Kano State (Kano Municipal, Nassarawa, Sabon-Gari, and Tarauni), and Ogun State (Ado-Odo Ota, Ifo, Obafemi-Owode, and Shagamu). These LGAs were purposively selected to represent the most densely populated urban and peri-urban areas where street and restaurant food vending is most prevalent.

This study forms part of the broader Nigeria Sodium Study (NaSS), which is a multi-faceted initiative designed to evaluate the country’s progress in implementing dietary sodium reduction policies [25,26,27,28,29]. The study employed a cross-sectional survey to examine the sodium content of commonly sold foods from street and restaurant food outlets in the three states of Nigeria between August 2024, and October 2024. The survey included a range of vendor types to reflect the diversity of Nigeria’s food retail landscape, such as street hawker stalls, self-service restaurants, quick-service restaurants (QSRs), and hotel restaurants. These vendors represent various culinary settings, from affordable, informal meals offered by hawkers to structured and more expensive options in hotels. This approach aimed to capture food sourcing and consumption patterns across different socioeconomic contexts.

### 2.1. Data Source

Food outlets were identified through convenience sampling in commercial areas within each selected LGA, targeting locations with high concentrations of food vendors such as markets, motor parks, busy roadsides, and commercial districts. Vendors were approached during peak meal times and invited to participate if they sold one or more foods from the pre-determined target list and agreed to participate. From each consenting vendor, research assistants collected samples of all available foods from a pre-determined target list. The study targeted 42 commonly consumed Nigerian dishes, determined a priori based on food frequency data from the Nigeria Sodium Study (NaSS) baseline dietary survey and expert consultation with nutritionists familiar with regional food practices. The target food list was developed using dietary recall and food frequency data from the Nigeria Sodium Study (NaSS) baseline survey [9], which identified the most commonly consumed street and restaurant foods among Nigerian adults. The final list prioritised high-frequency, high-sodium-contribution items, including rice-based dishes (e.g., jollof rice, rice and stew), legume-based foods (e.g., beans pottage), traditional soups and stews (e.g., egusi, efo riro, banga soup), and seasoned animal protein foods (e.g., suya, kilishi)—all identified as major sodium contributors in prior national dietary survey data [9]. To ensure completeness, the list was further validated through expert consultation with nutritionists with regional food knowledge. During fieldwork, no major commonly consumed foods were identified that were not already on the target list.

Data collection was conducted by twelve trained nutritionists and dietitians, four in the Federal Capital Territory, four in Kano State, and four in Ogun State, all with extensive experience in food and nutrition field surveys in Nigeria, including prior involvement in Nigeria Sodium Study data collection activities. All field staff followed a detailed field protocol encompassing a vendor approach script, standardised sampling procedures, salt meter operation guidelines, and real-time data capture using the FoodSwitch Data Collector App (The George Institute for Global Health, Sydney, Australia), to ensure systematic, consistent, and complete sample collection across all vendor types and survey periods. Prior to fieldwork, all staff completed a standardised refresher training, and pilot testing was conducted at a single site in each state to confirm operator proficiency before the main data collection commenced. Pilot tests were conducted to confirm operator proficiency. Two servings of each available target food were purchased from selected vendors at two separate meal periods (breakfast and lunch, where applicable). Sodium measurements from the two samples were averaged to obtain the final sodium concentration for each food item. Samples were carefully labelled, stored at ambient temperature in sealed containers, and transported to the testing site within 2 h of collection to maintain their integrity. Data on country, state, vendor name, vendor location, food name, food type, serving size, price, and food category (main meal, snack, or dessert) were recorded using the FoodSwitch Data Collector App, developed by The George Institute for Global Health, Australia. Trained research assistants used the app on smartphones to capture photographs of the food items and document detailed information. These photographs were uploaded to a secure cloud-based server for storage and later retrieved for data cleaning and analysis.

### 2.2. Sodium Concentration Measurement

Sodium content of food samples was primarily measured in situ using a portable ATAGO salt meter (ATAGO PAL-SALT, ATAGO Co. Ltd., Tokyo, Japan), following the manufacturer’s standard operating procedures. Portable salt meters have been used in food sodium assessment studies and have demonstrated acceptable utility for field-based estimation of sodium content in diverse food environments [30]. To ensure a representative sample and to reduce interference from the food matrix (such as fats and suspended solids), a 1:10 dilution protocol was followed [31,32,33,34]. Approximately 5.0 g of each food sample was weighed using a digital precision scale (±0.01 g) (SF-400 )and diluted with 45 mL of distilled water to achieve a total weight of 50.0 g. The mixture was homogenised using a Pocket Salt Meter (PAL-SALT, ATAGO Co., Ltd., Tokyo, Japan) until a uniform consistency was reached. A portion of the homogenised liquid was then applied to the sensor of the salt meter. To determine the actual sodium content, the raw salinity percentage recorded by the meter was adjusted using a dilution factor (DF) of 10. The final sodium concentration was calculated using the following equations: (1) Actual Salt Content (g/100 g) = Meter Reading (%) × 10; (2) Sodium Content (mg/100 g) = (Actual Salt Content (g)/2.5) × 1000 [30,31,32,35,36].

The ATAGO salt meter measures salinity in increments of 0.1%, which, after applying the dilution factor and conversion calculations, yields sodium concentrations in discrete numerical steps of 40 mg/100 g (e.g., 400.0, 440.0, 480.0 mg/100 g). Consequently, all reported sodium values reflect instrument resolution and calculation precision rather than investigator rounding.

### 2.3. Study Instrument Validation

To ensure data quality and assess measurement reliability, a random subsample of 10% of all collected food samples (*n* = 258) was selected for independent laboratory-based sodium analysis using flame photometry. Duplicate samples with identical identification codes were prepared, stored in sealed containers, and transported under cold chain conditions to an accredited analytical laboratory within hours of collection for sodium determination following standard analytical protocols.

Primary sodium measurements were obtained using the ATAGO salt meter. Laboratory measurements were conducted independently to field measurement results as a quality control measure to assess concordance between field-based and laboratory methods. Comparative data from both measurement methods were evaluated using Bland–Altman analysis to assess agreement between field-based ATAGO measurements and laboratory-based flame photometry results.

### 2.4. Statistical Analysis

Sodium concentrations of street and restaurant foods were analysed using descriptive and multilevel statistical approaches. Given the right-skewed distribution of sodium values, results are summarised using medians and interquartile ranges (IQRs). Sodium concentrations in food samples were classified into three categories based on established sodium content standards: low-sodium, medium-sodium, and high-sodium. Sodium categories (low ≤ 120 mg/100 g; medium > 120–600 mg/100 g; and high > 600 mg/100 g) followed cut-offs used in previous food composition and surveillance studies, including the United Kingdom Food Standards Agency traffic light classification [37,38].

Differences across Federal Capital Territory, Kano, and Ogun States were assessed using the Kruskal–Wallis H test, followed by Dunn’s test with Bonferroni correction for pairwise comparisons. Univariable analyses were first conducted to examine associations between sodium content and individual predictors. To assess differences in sodium content by food and vendor characteristics while accounting for clustering within testing centres (i.e., field measurement sites, one per state), a mixed-effects modelling framework was employed. Linear mixed-effects models were specified with food type, vendor type, and state included as fixed effects, reflecting substantive predictors of interest, while testing centres were included as random intercepts to account for non-independence of observations arising from shared collection and analytical centres.

Reference categories were selected a priori to support the interpretability and policy relevance of model estimates. Main meals were specified as the reference category for food type, as they constitute the primary component of daily dietary intake and contribute most substantially to overall sodium exposure. Street/hawker stalls were used as the reference category for vendor type, reflecting their prominence in the urban food environment and their relevance as a key source of prepared foods. Federal Capital Territory was specified as the reference category for state-level comparisons, serving as a neutral and policy-relevant benchmark as a major urban and administrative centre with a diverse food environment. Model assumptions were assessed through inspection of residual plots, and sodium concentrations were log-transformed to improve normality and homoscedasticity.

Fixed-effect estimates were exponentiated and expressed as percentage change relative to reference categories. Model-based estimated marginal means and 95% confidence intervals were obtained by back-transforming log-scale predictions, and the intraclass correlation coefficient (ICC) was calculated to quantify between-centre clustering. Overall fixed effects were assessed using Type III Wald tests with Satterthwaite-adjusted degrees of freedom. Observations with missing sodium values were excluded under a complete-case approach.

All analyses were conducted using R statistical software (version 4.4.0), and statistical significance was assessed at the two-sided 5% level.

## 3. Results

**Figure 1:**
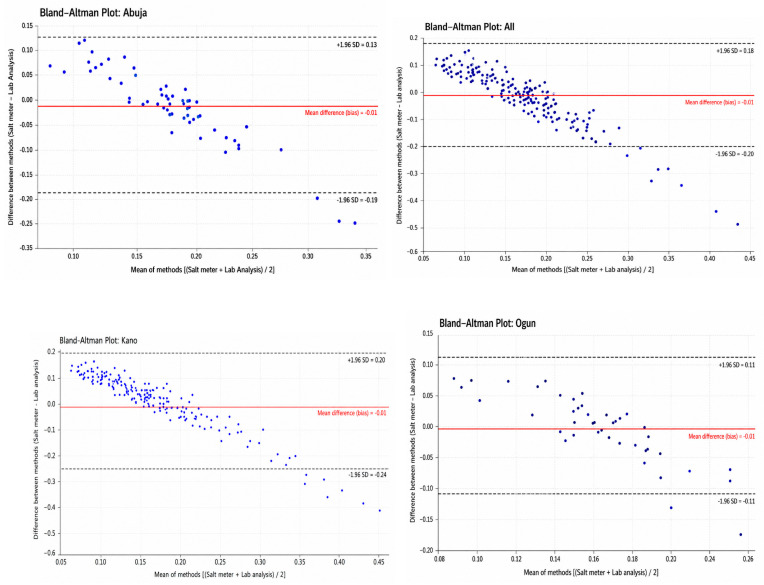
Bland-Altman Analysis of Agreement Between Field Salt Meter and Laboratory Sodium Measurements Across Study Sites.

The correlation between sodium concentrations measured using the ATAGO salt meter and laboratory analysis was weak (r = 0.23). Bland–Altman analysis showed a mean difference (bias) of −0.01, with limits of agreement ranging from −0.20 to 0.18 overall. Across study locations, mean biases were similarly small, although limits of agreement were widest in Kano (−0.24 to 0.20), followed by FCT (−0.19 to 0.17) and Ogun (−0.11 to 0.10) (Figure 1).

A total of 2583 street food samples were collected with the highest proportion obtained from the FCT (40.2%), followed by Ogun State (34.6%) and Kano State (25.2%). Across all states and survey areas, main meals were the predominant category of foods, accounting for between 62.1% and 88.1% of items, while snacks contributed a smaller share (2.7–21.3%) and desserts were the least sampled (4.4–15.6%). The proportion of snacks was notably higher in Kano State (overall 16.0%) compared with Federal Capital Territory (7.4%) and Ogun State (10.9%), whereas desserts were more prominent in selected urban LGAs in Kano such as Municipal (15.6%) and Ifo (11.9%) (Table 1).

Table 2 presents the distribution of sampled food types across the three states. A wide variety of foods were collected (n = 42), reflecting the diversity of the Nigerian street and restaurant food environment. Rice-based dishes were among the most commonly available, including rice and stew (7.8% in Federal Capital Territory, 4.5% in Kano, and 7.7% in Ogun) and jollof rice (7.2%, 5.1%, and 4.6% respectively). Legume-based foods were also prominent, particularly beans served with stew in Ogun State (7.9%). Geographic patterns in traditional soup availability reflected regional food preferences and the purposive selection of locally consumed foods, with bitterleaf soup more frequently sampled in Federal Capital Territory (4.9%), miyan kuka in Kano (1.5%), and vegetable-based soups such as ewedu (4.3%) and efo riro (2.5%) in Ogun State. Median food prices varied substantially across food types and states, with staple meals and traditional soups generally ranging from ₦800 to 2000 per serving (Supplementary Table S1).

Table 3 presents selected high-sodium foods across the three states. Soups consistently recorded the highest sodium concentrations, with ogbono soup (1360 mg/100 g), banga soup (1150 mg/100 g), and efo riro (1100 mg/100 g) among the highest in FCT; vegetable soup (1080 mg/100 g) and banga soup (1000 mg/100 g) in Kano; and banga soup (880 mg/100 g) in Ogun. Animal protein foods also showed elevated levels, with kilishi ranging from 620 to 1080 mg/100 g and suya from 240 to 920 mg/100 g across states. Staple dishes showed moderate concentrations, including jollof rice (640–680 mg/100 g) and beans pottage (670–760 mg/100 g). Snacks had lower but variable sodium content—chin chin ranged from 120 to 540 mg/100 g and puff puff from 160 to 600 mg/100 g across states. Overall median sodium content remained high across all states: 720 mg/100 g in FCT, 650 mg/100 g in Kano, and 600 mg/100 g in Ogun, indicating a consistently high-sodium food environment (Supplementary Table S2).

**Figure 2:**
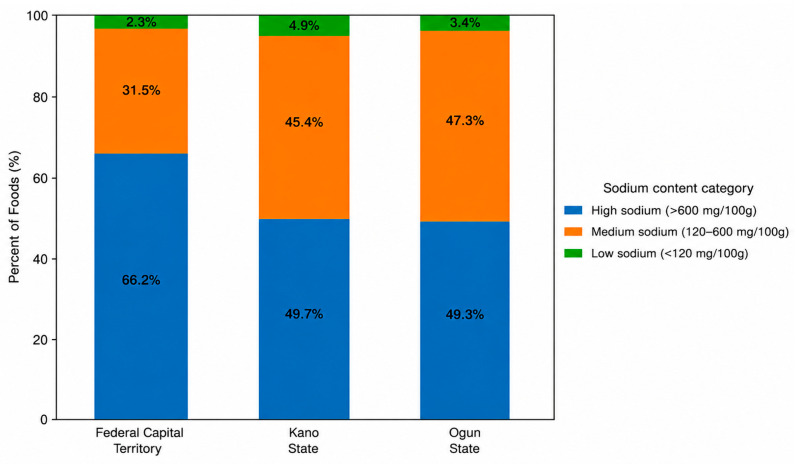
Distribution of street and restaurant foods by sodium content level in the Federal Capital Territory, Kano, and Ogun States, Nigeria.

Among the 2583 food samples, more than half of the foods were classified as high-sodium (>600 mg/100 g), accounting for 1431 samples (55.4%). Medium-sodium foods (120–600 mg/100 g) comprised 1045 samples (40.5%), while low-sodium foods (<120 mg/100 g) were least common, with 107 samples (4.1%). In the FCT (n = 1039), the majority of foods were classified as high-sodium (66.2%), followed by medium-sodium foods (31.5%), while low-sodium foods accounted for a small proportion (4.3%). In Kano State (n = 650), approximately half of the foods were high-sodium (49.7%), with a comparable proportion classified as medium-sodium (45.4%). Low-sodium foods represented 4.9%. Similarly, in Ogun State (n = 894), high-sodium foods comprised 49.3% of the samples, closely followed by medium-sodium foods (47.3%), while low-sodium foods were the least prevalent (3.4%, Figure 2).

Table 4 presents median sodium content by food category and vendor type across the three states. Differences in sodium concentrations across food categories were statistically significant in the FCT (*p* = 0.011) but not in Kano (*p* = 0.181) nor Ogun State (*p* = 0.877). Main meals generally had higher median sodium concentrations compared with snacks and desserts in the FCT and Kano, while less variation was observed across food categories in Ogun State. Differences across vendor types were statistically significant only in Ogun State (*p* < 0.001). Across states, restaurants/hotels generally exhibited higher median sodium concentrations than street/hawker stalls, although statistically significant differences by vendor type were observed only in Ogun State (*p* < 0.001).

**Figure 3:**
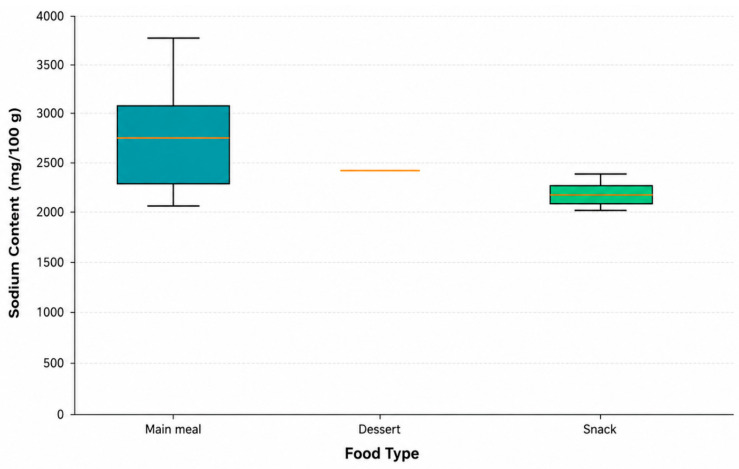
Sodium concentration (mg/100 g) in selected high-sodium foods by food type.

A small subset of foods exhibited exceptionally high sodium densities (>2000 mg/100 g). Among these foods, sodium concentrations in main meals ranged from 2060 mg/100 g to 3760 mg/100 g, with several observations exceeding 3000 mg/100 g. Within this subset, desserts recorded a sodium concentration of 2400 mg/100 g, while snacks ranged from 2020 mg/100 g to 2360 mg/100 g (Figure 3).

Supplementary Table S3 presents the results of the multilevel mixed-effects model assessing determinants of sodium content in the combined street and restaurant food environment across the Federal Capital Territory, Kano, and Ogun, with clustering at the testing-centre level. Descriptively, sodium concentrations varied across states, food categories, and vendor types. However, after adjustment for food type and vendor characteristics, state-level differences were no longer statistically significant (Type III ANOVA: F = 1.80, *p* = 0.217), despite sodium concentrations being descriptively lower in Kano (14.0% lower; *p* = 0.261) and Ogun (19.5% lower; *p* = 0.092) relative to the FCT.

Supplementary Table S3 presents the results of the multilevel mixed-effects model assessing determinants of sodium content in street and restaurant foods across the Federal Capital Territory, Kano, and Ogun, with clustering at the testing-centre level. Although sodium concentrations varied descriptively across states, food categories, and vendor types, state-level differences were not statistically significant after adjustment for food type and vendor characteristics (Type III Wald test: F = 1.80, *p* = 0.217), despite lower sodium concentrations in Kano (14.0% lower; *p* = 0.261) and Ogun (19.5% lower; *p* = 0.092) than in the FCT. Vendor type remained a significant predictor of sodium content (F = 3.90, *p* = 0.009), with hotel restaurants containing 15.4% higher sodium levels (*p* = 0.0006) and quick-service restaurants 21.9% lower sodium concentrations (*p* < 0.001) than street and hawker stalls, while self-service restaurants did not differ significantly (*p* = 0.201). Food type was the strongest determinant of sodium content (F = 45.14, *p* < 0.001), with snacks and desserts containing 29.3% and 34.1% lower sodium concentrations, respectively, than main meals (both *p* < 0.001). Effect-size estimates showed a small effect of food type (ω^2^ = 0.033), a negligible effect of vendor type (ω^2^ = 0.003), and modest between-centre clustering (ICC = 0.07). Overall, these findings indicate that variation in sodium content was driven primarily by food category and vendor environment rather than geographic location.

## 4. Discussion

This study provides a comprehensive assessment of sodium content in street and restaurant foods across multiple Nigerian settings and presents both descriptive and model-adjusted evidence of population exposure to dietary sodium in the Federal Capital Territory (FCT), Kano State, and Ogun State. The findings demonstrate that sodium levels in commonly consumed foods are consistently high and structurally patterned across food categories and vendor environments. Importantly, adjusted analyses showed that variation in sodium exposure was driven primarily by food type and vendor context rather than geographic location, suggesting that sodium exposure is embedded within prevailing food system practices rather than being place-specific. This pattern likely reflects the central role of salt, bouillon cubes, seasonings, and processed condiments in everyday Nigerian cooking, alongside the widespread consumption of street and restaurant foods. Given that these foods constitute a major component of daily dietary intake for many households, they are likely to contribute substantially to excessive sodium intake and population-level cardiovascular disease risk [9].

Placing these findings in an international context, the sodium levels observed in this study show both similarities and differences compared with evidence from a street food study in Malaysia [38]. In the current analysis, main meals consistently exhibited the highest median sodium concentrations across study locations, whereas snacks and desserts showed greater variation across states. In contrast, the Malaysian study reported the highest sodium levels among snack-type street foods, followed by main meals and desserts, with processed snack items reaching particularly high sodium concentrations (approximately 452–584 mg/100 g). These differences likely reflect variation in food preparation methods, ingredient use, consumer preferences, and broader food environments between settings. While Malaysian street food snacks appear to be more sodium-dense due to greater reliance on processed and reformulated products [38], Nigerian street and restaurant foods, particularly main meals, may contain higher sodium levels because of discretionary salt use, seasoning practices, and condiment-based cooking methods [9,24,25,39]. Overall, these findings highlight the need for context-specific sodium reduction strategies that account for dominant dietary patterns, cooking practices, and informal food environments.

Furthermore, beyond Malaysia, the sodium levels documented in this study are broadly consistent with findings from street food assessments conducted across several LMICs, although the dominant food categories and sources of sodium vary by context. Studies from Mozambique and Argentina have reported high sodium concentrations in commonly consumed cooked and artisanal street and restaurant foods, with many single items contributing a substantial proportion of recommended daily sodium intake [40,41], mirroring the elevated sodium concentrations observed in Nigerian main meals and soups. Evidence from Ghana, South Africa, and the Nigeria Sodium Study has similarly demonstrated high population sodium exposure and identified salt, bouillon cubes, and seasonings as major contributors to dietary sodium intake [39,42]. Studies from China and parts of Latin America have also reported substantial sodium contributions from packaged, takeaway, and semi-processed foods, with some individual portions exceeding 1000 mg sodium per serving [40,43]. While the relative contribution of sodium sources varies across settings, the Nigerian findings suggest that discretionary salt use, seasoning practices, and condiment-based cooking methods remain important drivers of sodium exposure alongside the growing influence of processed and prepared foods. Collectively, these cross-country comparisons highlight that elevated sodium content in street and restaurant foods is a shared global challenge, although the relative importance of different sodium sources may vary across food systems, reinforcing the need for context-specific sodium reduction strategies [11,44].

Our findings support the need for stronger implementation of population-wide sodium reduction strategies recommended by the WHO and World Health Assembly, particularly interventions targeting food environments and upstream drivers of sodium exposure [45]. The consistently high sodium concentrations observed across commonly consumed street and restaurant foods, especially among main meals and foods from hotel restaurant settings, suggest that sodium exposure in Nigeria is structurally embedded within food preparation and vending practices rather than driven solely by individual consumer behaviour. Although sodium concentrations differed by vendor type, elevated sodium levels were observed across both street and restaurant settings, indicating that excessive sodium is a system-wide characteristic of Nigeria’s prepared food environment.

Our results also support the need for implementation of key components of the WHO SHAKE technical package [15], particularly strengthening sodium surveillance, improving food environments, supporting reformulation and sodium reduction in prepared foods, and increasing public awareness of salt-related health risks. The findings further highlight an important policy gap within Nigeria’s sodium reduction efforts. While Nigeria’s National Multisectoral Action Plan (NMAP) for NCD prevention and the National Sodium Reduction Guidelines for Packaged Foods provide direction for industrially processed foods [13,16], equivalent sodium guidance and reformulation strategies for street foods, restaurant foods, and informal food vendors remain limited. Given the high sodium concentrations observed across commonly consumed street and restaurant foods in this study, extending sodium reduction policies beyond packaged foods to informal and prepared food environments will be important for achieving national and global sodium reduction targets [46]. Beyond structural and regulatory interventions, public education and behaviour change communication are essential complements to food environment strategies. Studies have highlighted the multifaceted and culturally embedded role of salt in Nigerian and broader African culinary practices, where it is valued as a flavour enhancer, preservative, and marker of culinary skill, alongside widespread knowledge gaps regarding recommended daily sodium intake [9,25,42]. These findings highlight the need for sodium reduction messages that are culturally appropriate, positively framed, and delivered through trusted community channels. Extending such communication to African diaspora communities, where traditional West African cooking practices remain common, could further amplify the public health impact of sodium reduction efforts beyond the continent [25].

More broadly, the findings demonstrate how urban food environments and informal food systems can shape population-level nutrition risks, reinforcing the importance of multisectoral food-system interventions for improving dietary health and supporting progress toward Sustainable Development Goals related to health, nutrition, and sustainable urban food systems [47,48].

From a public health perspective, these findings have important implications for nutrition policy and the prevention of NCDs. The observation that sodium concentrations were driven primarily by food type and vendor environment suggests that sodium exposure is shaped largely by food preparation practices and characteristics of the broader food environment rather than individual consumer behaviour alone. Consequently, interventions targeting individual behaviour alone are unlikely to achieve meaningful reductions at the population level. Instead, coordinated regulatory, environmental, and system-level strategies are needed, including reformulation of commonly consumed foods, establishment of sodium standards across food categories, and guidance for food preparation practices among different vendor types [49,50,51]. These findings also highlight the importance of engaging food vendors, informal food systems, and the broader food service sector in national sodium reduction efforts.

### Limitations

Several limitations should be considered when interpreting these findings. As part of the study validation process, sodium measurements obtained using the ATAGO salt meter were compared with laboratory-based flame photometry, and some disagreement between methods was observed. While laboratory analysis is commonly considered a reference standard, discrepancies in sodium estimation within complex mixed-food matrices have been reported in similar field-based sodium assessments. Given the operational suitability of the ATAGO salt meter for large-scale field-based sodium assessment and its previous application in diverse food environments [30,31,32,35,36], ATAGO measurements were retained as the primary analytical data source. Although laboratory-based methods such as flame photometry or inductively coupled plasma optical emission spectrometry (ICP-OES) are generally regarded as reference methods for sodium determination because of their higher analytical accuracy and precision, their application to all 2583 food samples was not feasible owing to the substantial cost, specialised laboratory infrastructure, longer processing time, and logistical challenges associated with transporting perishable food samples across multiple field sites. The ATAGO salt meter was therefore selected as a pragmatic field-based method that enabled rapid, standardised, and large-scale sodium assessment under real-world conditions. Nevertheless, the absence of certified reference standards for all tested foods and the observed disagreement between methods suggest that some degree of measurement error remains possible.

The analysis focused on sodium content per 100 g of food and did not directly link sodium concentrations to individual food consumption patterns. Consequently, although the study identified foods with high sodium content, it was not possible to quantify the contribution of specific street and restaurant foods to overall dietary sodium intake. Food sampling was also conducted within study budget constraints, which limited the range of foods that could be included. In addition, the study focused on a pre-specified list of commonly consumed foods identified through the Nigeria Sodium Study dietary survey. As this list contained a greater proportion of main meals than snacks or desserts, the observed distribution of sampled food categories may partly reflect the composition of the target food list, although the selected foods were intended to represent the most frequently consumed foods in the study settings. Although hotel restaurants were sampled and frequently demonstrated high sodium concentrations, higher-cost specialty dishes and premium food outlets were underrepresented. Consequently, the findings primarily reflect foods that were affordable and accessible within the study’s sampling framework, and sodium concentrations in more expensive food environments may have been underestimated. The 2 h transport protocol and duplicate averaging approach, while consistent with comparable field-based studies, are not universally standardised. Some studies have employed immediate on-site measurement or refrigerated transport, and such differences in handling may contribute to inter-study variability in sodium estimates.

Despite these limitations, this study has several important strengths and novel contributions. The integration of large-scale field-based sodium assessment across multiple sites with multilevel mixed-effects modelling enabled both population-level characterization of sodium exposure and assessment of structural determinants of sodium content. The combined use of descriptive epidemiology and model-adjusted estimated marginal means facilitated identification of systemic drivers of sodium exposure across food types and vendor environments rather than isolated high-sodium foods alone. The large sample size, inclusion of centre-level random effects, and multilevel analytical framework strengthen the robustness, internal validity, and policy relevance of the findings. To our knowledge, this represents one of the most comprehensive assessments of sodium content in street and restaurant foods conducted in Sub-Saharan Africa.

## 5. Conclusions

This study demonstrates that sodium concentrations in commonly consumed street and restaurant foods in Nigeria are high, with more than half of the sampled foods classified as high-sodium. Variation in sodium content was driven primarily by food type and vendor environment rather than geographic location, highlighting the importance of food-system factors in shaping sodium exposure. Reducing sodium intake will require coordinated policy and food-environment interventions, including vendor engagement, sodium reduction in prepared foods as part of broader national sodium reduction efforts.

## Figures and Tables

**Table 1:** Availability of street and restaurant foods by state, area and food category in Nigeria.

States	Areas	Main Meals n (%)	Snacks n (%)	Desserts n (%)	Total n (%)
Federal Capital Territory	Federal Capital Territory Municipal Area Council	237 (82.3)	29 (10.1)	22 (7.6)	288 (27.7)
	Gwagwalada	223 (79.6)	28 (10.0)	29 (10.4)	280 (26.9)
	Kuje	199 (88.1)	17 (7.5)	10 (4.4)	226 (21.8)
	Bwari	196 (80.0)	24 (9.8)	25 (10.2)	245 (23.6)
Kano	Sabon-gari	154 (67.0)	49 (21.3)	27 (11.7)	230 (35.4)
	Kano Municipal	123 (76.9)	12 (7.5)	25 (15.6)	160 (24.6)
	Tarauni	110 (82.1)	14 (10.4)	10 (7.5)	134 (20.6)
	Nassarawa	94 (74.6)	20 (15.9)	12 (9.5)	126 (19.4)
Ogun	Ado-Odo Ota	132 (75.9)	23 (13.2)	19 (10.9)	174 (20.0)
	Obafemi-Owode	218 (83.5)	19 (7.3)	24 (9.2)	261 (30.0)
	Ifo	238 (76.9)	18 (5.8)	19 (6.1)	275 (31.6)
	Shagamu	105 (65.6)	31 (19.4)	24 (15.0)	160 (18.4)

% for main meals, snacks, and desserts are calculated within each area (row percentages). % in the Total column are calculated relative to the total number of samples collected within each state.

**Table 2:** Distribution of commonly consumed street and restaurant foods across the Federal Capital Territory, Kano, and Ogun States.

Food Category	Food Name	Federal Capital Territory n (%)	Kano n (%)	Ogun n (%)
Main meals/Mixed dishes	Abacha and ugba	0 (0.0)	2 (0.3)	1 (0.1)
	Akara (bean cake)	26 (2.5)	23 (3.5)	20 (2.2)
	Beans and rice	47 (4.5)	26 (4.0)	62 (6.9)
	Beans (served with stew)	27 (2.6)	20 (3.1)	71 (7.9)
	Beans pottage	57 (5.5)	12 (1.8)	16 (1.8)
	Cooked indomie noodles with vegetables and/or fried egg	15 (1.4)	18 (2.8)	23 (2.6)
	Egg sauce	14 (1.3)	22 (3.4)	17 (1.9)
	Fried yam, plantain, potatoes & pepper sauce	38 (3.7)	51 (7.8)	33 (3.7)
	Jollof rice	75 (7.2)	33 (5.1)	41 (4.6)
	Jollof spaghetti	19 (1.8)	21 (3.2)	23 (2.6)
	Masa (waina)	17 (1.6)	39 (6.0)	2 (0.2)
	Moin moin	27 (2.6)	24 (3.7)	24 (2.7)
	Opa	22 (2.1)	15 (2.3)	9 (1.0)
	Rice and stew	81 (7.8)	29 (4.5)	69 (7.7)
	Tuwo shinkafa	18 (1.7)	3 (0.5)	5 (0.6)
Soups and stews	Afang soup	24 (2.3)	0 (0.0)	21 (2.3)
	Banga soup	20 (1.9)	3 (0.5)	5 (0.6)
	Bitterleaf soup	51 (4.9)	10 (1.5)	15 (1.7)
	Edikakong soup	8 (0.8)	0 (0.0)	12 (1.3)
	Efo riro	18 (1.7)	2 (0.3)	22 (2.5)
	Egusi soup	64 (6.2)	24 (3.7)	56 (6.3)
	Ewedu soup (with stew/beans)	16 (1.5)	9 (1.9)	38 (4.3)
	Miyan kuka soup	21 (2.0)	10 (1.5)	0 (0.0)
	Ogbono soup	13 (1.3)	6 (0.9)	23 (2.6)
	Oha soup	14 (1.3)	6 (0.9)	13 (1.5)
	Okra soup	38 (3.7)	26 (4.0)	21 (2.3)
	Pepper soup	7 (0.7)	5 (0.8)	12 (1.3)
	Vegetable soup	38 (3.7)	27 (4.2)	41 (4.6)
	White soup	23 (2.2)	3 (0.5)	5 (0.6)
Snacks/baked foods	Burabisco	15 (1.4)	5 (0.8)	0 (0.0)
	Chin chin	18 (1.7)	27 (4.2)	22 (2.5)
	Cookies	2 (0.2)	6 (0.9)	13 (1.5)
	Doughnut	20 (1.9)	24 (3.7)	28 (3.1)
	Meat pie	20 (1.9)	32 (4.9)	22 (2.5)
	Puff puff	22 (2.1)	27 (4.2)	29 (3.2)
Animal protein/fast foods	Burgers	5 (0.5)	4 (0.6)	8 (0.9)
	Kilishi	21 (2.0)	7 (1.1)	12 (1.3)
	Shawarma	11 (1.1)	5 (0.8)	9 (1.0)
	Suya	12 (1.2)	9 (1.4)	3 (0.3)
Desserts/accompaniments	Cakes	16 (1.5)	12 (1.8)	16 (1.8)
	Coleslaw (mayo dressing)	21 (2.0)	9 (1.4)	23 (2.6)
	Roasted plantain & pepper sauce (bole)	18 (1.7)	14 (2.2)	21 (2.3)
Total		1039 (40.2)	650 (25.2)	894 (34.6)

**Table 3:** Median (IQR) Sodium Content of Selected High-Sodium Foods by State (mg/100 g) in the Federal Capital Territory, Kano, and Ogun States.

Food	FCT Median (IQR)	Kano Median (IQR)	Ogun Median (IQR)
Ogbono soup	1360 (810)	440 (610)	680 (410)
Banga soup	1150 (665)	1000 (0)	880 (560)
White soup	1120 (240)	–	280 (480)
Efo riro	1100 (520)	–	380 (600)
Vegetable soup	950 (440)	1080 (640)	520 (460)
Jollof rice	640 (240)	640 (250)	680 (480)
Beans pottage	760 (160)	740 (230)	670 (350)
Cooked Indomie noodles with vegetables and/or fried egg	800 (220)	600 (455)	480 (560)
Kilishi	1080 (660)	880 (640)	620 (285)
Suya	920 (940)	560 (460)	240 (0)
Chin chin	180 (400)	120 (120)	540 (480)
Puff puff	160 (150)	360 (240)	600 (410)
All foods	720 (480)	650 (520)	600 (440)

Selected foods represent examples of foods with relatively high sodium content within the study. Complete food-specific sodium results are presented in Supplementary Table S2.

**Table 4:** Median Sodium Content of Street Food Categories and Vendor Types Across the Federal Capital Territory, Kano, and Ogun States, Nigeria.

	Federal Capital Territory (n = 1039)		Kano (n = 650)		Ogun (n = 894)	
Category	n (%)	Median (IQR) (mg/100 g)	n (%)	Median (IQR) (mg/100 g)	n (%)	Median (IQR) (mg/100 g)
Street and restaurant foods category						
Main meals	855 (82.3)	760.0 (440.0) ^a^	481 (74.0)	680.0 (280.0)	716 (80.4)	600.0 (440.0)
Snacks	98 (9.4)	440.0 (530.0)	95 (14.6)	360.0 (240.0)	92 (10.3)	600.0 (405.0)
Desserts	86 (8.3)	440.0 (440.0)	74 (11.4)	320.0 (325.0)	83 (9.3)	640.0 (360.0)
*p*-value		0.011		0.181		0.877
Food vendor types						
Street/hawker stalls	206 (19.8)	680.0 (445.0)	307 (47.2)	600.0 (280.0)	98 (11.0)	560.0 (520.0)
Restaurants/Hotels	392 (37.7)	760.0 (540.0)	88 (13.5)	680.0 (240.0)	108 (12.1)	660.0 (425.0)
Quick-service restaurants	372 (35.8)	720.0 (55.0)	211 (32.5)	560.0 (280.0)	551 (61.6)	600.0 (440.0)
Self-service restaurants	69 (6.6)	640.0 (320.0)	44 (6.8)	600.0 (280.0)	137 (15.3)	640.0 (400.0)
*p*-value		0.170		0.140		<0.001

^a^ Statistically significant differences between categories based on Dunn’s post -hoc test with Bonferroni correction (*p* < 0.05).

## Data Availability

The data that support the findings of this study are available from the George Institute for Global Health (TGI) Database; however, restrictions apply to their availability, as the data were used under license for the current study and are therefore not publicly available. The data are, however, available from the authors upon reasonable request and with permission of the Nigeria Sodium Study team.

## Supplementary Materials

The following supporting information can be downloaded at https://www.mdpi.com/article/10.3390/nu18142348/s1, Table S1. Distribution and Median Prices of Commonly Consumed Street and Cooked Foods Across the Federal Capital Territory, Kano, and Ogun States; Table S2. Median Sodium Content of Commonly Consumed Foods by State (Federal Capital Territory, Kano, and Ogun), Nigeria; Table S3. Multilevel Mixed-Effects Model of Sodium Content in Street Foods in Abuja, Kano, and Ogun States.

## Abbreviations

The following abbreviations are used in this manuscript:

CVD	Cardiovascular Disease
FMoH	Federal Ministry of Health
GBD	Global Burden of Disease
LMICs	Low- and Middle-Income Countries
NaCl	Sodium Chloride
NaSS	Nigeria Sodium Study
NCDs	Non-Communicable Diseases
NMSAP	National Multisectoral Action Plan
QSR	Quick-Service Restaurant
SDG	Sustainable Development Goals
SHAKE	Surveillance, Harness Industry, Adopt Standards, Knowledge, Environment
WHO	World Health Organisation
