# Supplementary material for: Sodium Content of Street and Restaurant Foods in Nigeria: A Multilevel Analysis of Food System Determinants
Source: Nutrients. 2026 Jul 17;18(14):2348. doi: 10.3390/nu18142348 (PMC13415127; doi:10.3390/nu18142348)
Supplement: Supplementary file 1 [file nutrients-18-02348-s001.zip › nutrients-4416033-supplementary.pdf]

Supplementary Table S1. Distribution and Median Prices of Commonly Consumed Street and Cooked Foods Across the Federal Capital Territory, Kano, and Ogun States

Food Name	Federal Capital Territory n (%)	Median Price (IQR)	Kano n (%)	Price (IQR)	Ogun n (%)	Price (IQR)
Abacha and ugba	0 (0)	-	2 (0.3)	1000 (0)/serve	1 (0.1)	1000 (1000)/serve
Afang soup	24 (2.3)	1500 (1125)/serve	0 (0.0)	0 (0)/serve	21 (2.3)	1000 (1050)/serve
Akara (bean cake)	26 (2.5)	400 (600)/piece	23 (3.5)	500 (500)/piece	20 (2.2)	600 (800)/pieces
Banga soup	20 (1.9)	1000 (500)/serve	3 (0.5)	2000 (0)/serve	5 (0.6)	1000 (750)/serve
Beans and rice	47 (4.5)	1000 (250)/serve	26 (4.0)	1000 (350)/serve	62 (6.9)	1000 (600)/serve
Beans pottage	57 (5.5)	800 (600)/serve	12 (1.8)	1000 (1000)/serve	16 (1.8)	1000 (500)/serve
Beans, brown, cooked from dried (served with stew)	27 (2.6)	800 (300)/serve	20 (3.1)	1000 (500)/serve	71 (7.9)	1000 (275)/serve
Bitterleaf soup	51 (4.9)	1000 (300)/serve	10 (1.5)	2000 (1250)/serve	15 (1.7)	1000 (1150)/serve
Burabisco	15 (1.4)	1000 (200)/serve	5 (0.8)	1000 (1000)/serve	0 (0.0)	0 (0)/serve
Burgers	5 (0.5)	3000 (1500)/piece	4 (0.6)	3250 (2000)/piece	8 (0.9)	2450 (4250)/piece
Cakes	16 (1.5)	700 (700)/piece	12 (1.8)	1000 (575)/piece	16 (1.8)	1000 (1000)/piece
Chin chin	18 (1.7)	500 (700)/pack	27 (4.2)	500 (575)/pack	22 (2.5)	700 (500)/pack
Coleslaw, "mayo" dressing, plain	21 (2.0)	500 (825)/serve	9 (1.4)	1000 (900)/serve	23 (2.6)	1000 (800)/serve
Cookies	2 (0.2)	850 (0)/pack	6 (0.9)	1000 (250)/pack	13 (1.5)	1000 (1600)/pack
Cooked indomie noodles with vegetables and/or fried egg	15 (1.4)	1200 (825)/serve	18 (2.8)	1500 (550)/serve	23 (2.6)	1000 (500)/serve
Doughnut	20 (1.9)	500 (575)/piece	24 (3.7)	1000 (475)/piece	28 (3.1)	800 (700)/piece
Edikakong soup	8 (0.8)	1600 (575)/serve	0 (0.0)	0 (0)/serve	12 (1.3)	1000 (1000)/serve
Efo riro	18 (1.7)	1400 (800)/serve	2 (0.3)	1750 (0)/serve	22 (2.5)	1000 (1100)/serve

Egg sauce	14 (1.3)	900 (500)/serve	22 (3.4)	1000 (553)/serve	17 (1.9)	1000 (488)/serve
Egusi soup	64 (6.2)	1000 (650)/serve	24 (3.7)	1500 (1000)/serve	56 (6.3)	1000 (950)/serve
Ewedu soup with, stew and beans soup	16 (1.5)	1100 (775)/serve	9 (1.9)	1500 (750)/serve	38 (4.3)	1000 (1000)/serve
Fried yam, plantain, potatoes & pepper sauce	38 (3.7)	500 (600)/piece	51 (7.8)	1000 (700)/piece	33 (3.7)	1000 (763)/piece
Jollof rice	75 (7.2)	1000 (800)/serve	33 (5.1)	1500 (950)/serve	41 (4.6)	1000 (800)/serve
Jollof spaghetti	19 (1.8)	1400 (900)/serve	21 (3.2)	1500 (1000)/serve	23 (2.6)	1500 (1000)/serve
Kilishi	21 (2.0)	1000 (500)/pack	7 (1.1)	2000 (500)/pack	12 (1.3)	1750 (1850)/pack
Masa (waina)	17 (1.6)	400 (350)/piece	39 (6.0)	1000 (800)/piece	2 (0.2)	1000 (0)/piece
Meatpie	20 (1.9)	950 (650)/piece	32 (4.9)	1000 (500)/piece	22 (2.5)	800 (400)/piece
Miyan kuka soup	21 (2.0)	400 (850)/serve	10 (1.5)	1000 (0)/serve	0 (0.0)	1000 (1200)/serve
Moin moin	27 (2.6)	300 (350)/piece	24 (3.7)	1000 (500)/piece	24 (2.7)	1000 (500)/piece
Ogbono soup	13 (1.3)	1100 (275)/serve	6 (0.9)	2250 (1600)/serve	23 (2.6)	1000 (700)/serve
Oha soup	14 (1.3)	1600 (650)/serve	6 (0.9)	2000 (676)/serve	13 (1.5)	1000 (500)/serve
Okra soup	38 (3.7)	1000 (700)/serve	26 (4.0)	1000 (200)/serve	21 (2.3)	1000 (650)/serve
Opa	22 (2.1)	300 (325)/piece	15 (2.3)	500 (350)/piece	9 (1.0)	500 (450)/piece
Pepper soup	7 (0.7)	2500 (1125)/serve	5 (0.8)	2000 (1500)/serve	12 (1.3)	1400 (1000)/serve
Puff puff	22 (2.1)	200 (300)/piece	27 (4.2)	1000 (500)/piece	29 (3.2)	800 (550)/pieces
Rice and stew (boiled with salt)	81 (7.8)	1000 (700)/serve	29 (4.5)	1500 (1000)/serve	69 (7.7)	1000 (525)/serve
Roasted plantain & pepper sauce (bole)	18 (1.7)	1000 (450)/piece	14 (2.2)	1000 (425)/piece	21 (2.3)	600 (550)/piece
Shawarma	11 (1.1)	2900 (500)/piece	5 (0.8)	2250 (1175)/piece	9 (1.0)	1750 (1400)/piece

Suya	12 (1.2)	1000 (150)/serve	9 (1.4)	1500 (1000)/serve	3 (0.3)	500 (0)/serve
Tuwo shinkafa	18 (1.7)	500 (750)/serve	3 (0.5)	2000 (0)/serve	5 (0.6)	800 (500)/serve
Vegetable soup	38 (3.7)	1000 (700)/serve	27 (4.2)	1500 (875)/serve	41 (4.6)	1000 (1000)/serve
White soup	23 (2.2)	2000 (1500)/serve	3 (0.5)	2500 (0)/serve	5 (0.6)	1500 (1075)/serve

Supplementary Table S2. Median (QR) Sodium Content of Commonly Consumed Foods by State (Federal Capital Territory, Kano, and Ogun), Nigeria

**Food Name**	**Federal Capital Territory (Median ± IQR) Mg/100g**	**Kano (Median ± IQR) Mg/100g**	**Ogun (Median ± IQR) Mg/100g**
Abacha and ugba	-	420.0 ± 0.0	-
Afang soup	880.0 ± 320.0	-	640.0 ± 450.0
Akara (bean cake)	660.0 ± 200.0	680.0 ± 320.0	740.0 ± 565.0
Banga soup	1150.0 ± 665.0	1000.0 ±0.0	880.0 ± 560.0
Beans and rice	680.0 ± 780.0	460.0 ± 460.0	540.0 ± 450.0
Beans pottage	760.0 ± 160.0	740.0 ± 230.0	670.0 ± 350.0
Beans, brown, cooked from dried (served with stew)	860.0 ± 600.0	760.0 ± 500.0	580.0 ± 480.0
Bitterleaf soup	1060.0 ± 360.0	700.0 ± 280.0	600.0 ± 480.0
Burgers	600.0 ± 80.0	520.0 ± 140.0	740.0 ± 550.0
Burabisco	560.0 ± 380.0	520.0 ± 420.0	
Cakes	450.0 ± 110.0	340.0 ± 80.0	640.0 ± 520.0
Chin chin	180.0 ± 400.0	120.0 ± 120.0	540.0 ± 480.0
Coleslaw, “mayo” dressing, plain	260.0 ± 100.0	300.0 ± 80.0	760.0 ± 400.0
Cooked Indomie noodles with vegetables and/or fried egg	800.0 ± 220.0	600.0 ± 455.0	480.0 ± 560.0
Cookies	240.0	260.0 ± 990.0	640.0 ± 200.0
Doughnut	360.0 ± 370.0	320.0 ± 155.0	660.0 ± 480.0
Edikakong soup	810.0 ± 195.0	-	540.0 ± 445.0
Efo riro	1100.0 ± 520.0	-	380.0 ± 600.0
Egg sauce	1080.0 ± 590.0	900.0 ± 425.0	620.0 ± 250.0
Egusi soup	860.0 ± 290.0	1100.0 ± 520.0	600.0 ± 360.0
Ewedu soup with stew and beans soup	1000.0 ± 460.0	1240.0 ± 310.0	620.0 ± 510.0
Fried yam, plantain, potatoes & pepper sauce	380.0 ± 160.0	480.0 ± 480.0	620.0 ± 350.0
Jollof rice	640.0 ± 240.0	640.0 ± 250.0	680.0 ± 480.0
Jollof spaghetti	600.0 ± 220.0	560.0 ± 320.0	640.0 ± 520.0
Kilishi	1080.0 ± 660.0	880.0 ± 640.0	620.0 ± 285.0
Masa (waina)	40.0 ± 190.0	300.0 ± 320.0	-
Meatpie	440.0 ± 445.0	470.0 ± 325.0	540.0 ± 440.0
Miyan kuka soup	1000.0 ± 430.0	1060.0 ± 510.0	-

Moin moin	680.0 ± 120.0	920.0 ± 240.0	540.0 ± 550.0
Ogbono soup	1360.0 ± 810.0	440.0 ± 610.0	680.0 ± 410.0
Oha soup	1050.0 ± 665.0	920.0 ± 330.	660.0 ± 500.0
Okra soup	770.0 ± 200.0	1000.0 ± 410.0	640.0 ± 430.0
Opa	680.0 ± 130.0	780.0 ± 240.0	680.0 ± 500.0
Pepper soup	1000.0 ± 400.0	760.0 ± 500.0	560.0 ± 280.0
Puff puff	160.0 ± 150.0	360.0 ± 240.0	600.0 ± 410.0
Rice and stew (boiled with salt)	680.0 ± 370.0	440.0 ± 420.0	600.0 ± 400.0
Roasted plantain & pepper sauce (bole)	500.0 ± 360.0	630.0 ± 540.0	680.0 ± 200.0
Shawarma	520.0 ± 240.0	440.0 ± 120.0	680.0 ± 200.0
Suya	920.0 ± 940.0	560.0 ± 460.0	240.0
Tuwo shinkafa	0.0 ± 0.0	-	880.0 ± 800.0
Vegetable soup	950.0 ± 440.0	1080.0 ± 640.0	520.0 ± 460.0
White soup	1120.0 ± 240.0	-	280.0 ± 480.0
**Total**	**720.0 ± 480.0**	**650.0 ± 520.0**	**600.0 ± 440.0**

Supplementary Table S3. Multilevel mixed-effects model of sodium content in street foods in Federal Capital Territory, Kano and Ogun States

Model component	Variable	Category	Estimate (β)	SE	% Change†	p-value
**Fixed effects**	**State**	Federal Capital Territory (Ref)	Ref	Ref	Ref	—
		Kano	−0.151	0.127	−14.0	0.261
		Ogun	−0.248	0.133	−22.0	0.092
	**Vendor type**	Street/hawker stall (Ref)	Ref	Ref	Ref	—
		Restaurants/hotels	0.144	0.042	+15.5	<0.001*
		Self-service restaurants	0.068	0.053	+7.0	0.201
		Quick-service restaurants (QSR)	−0.217	0.045	−19.5	<0.001*
	**Food type**	Main meal (Ref)	Ref	Ref	Ref	—
		Snack	−0.346	0.042	−29.3	<0.001*
		Dessert	−0.417	0.045	−34.1	<0.001*
**Random effects**	Centre (intercept)	—	Variance = 0.033	SD = 0.182	—	—
	Residual	—	Variance = 0.436	SD = 0.660	—	—
**Model-level tests**	State	—	—	—	—	*F* = 1.80, *p* = 0.217
	Vendor type	—	—	—	—	*F* = 3.90, *p* = 0.009*
	Food type	—	—	—	—	*F* = 45.14, *p* < 0.001*
**Effect sizes**	State (Kruskal–Wallis)	—	—	—	$\varepsilon^2 < 0.001$	Negligible
	Vendor type	—	—	—	$\omega^2 = 0.003$	Negligible
	Food type	—	—	—	$\omega^2 = 0.033$	Small
	Between-centre clustering	—	—	—	ICC = 0.07	7% of variance
	Marginal R^2	—	—	—	0.039	Fixed effects
	Conditional R^2	—	—	—	0.109	Fixed + random effects

ICC = intraclass correlation coefficient; ε^2 = epsilon-squared; ω^2 = omega-squared; R^2 = coefficient of determination.
